# F110. THE BRIEF NEGATIVE SYMPTOM SCALE (BNSS): VALIDATION IN A MULTICENTER BRAZILIAN STUDY

**DOI:** 10.1093/schbul/sby017.641

**Published:** 2018-04-01

**Authors:** Heydrich Medeiros, Helio Elkis, Selene Vasconcelos, Diana Martins, Raissa Alexandria, Ana Albuquerque, Rosana Freitas, Maria Scardoelli, Elaine Di Sarno, Isabel Napolitano, Graça Oliveira, Adriana Vizzotto, Antônio Peregrino, Murilo Lima

**Affiliations:** 1 Federal University of Paraíba; 2 University of Sao Paulo Medical School; 3 University of Pernambuco; 4 University of São Paulo

## Abstract

**Background:**

Negative symptoms are a core feature of schizophrenia. The Brief Negative Symptom Scale (BNSS) is a new scale developed to assess negative symptoms in schizophrenia.

**Methods:**

The present study aimed to examine the construct validity of BNSS, by using convergent and divergent validities as well as factor analysis, in a Brazilian sample of 111 outpatients diagnosed with schizophrenia by DSM-5. Patients were evaluated by the Brazilian version of the BNSS and positive and negative subscales of the Positive and Negative Syndrome Scale (PANSS)

**Results:**

Assessment of patients by both instruments revealed an either an excellent internal consistency (Cronbach’s alpha = 0.938) or inter-rater reliability (ICC = 0.92), as well as a strong correlation between BNSS and negative PANSS (r = 0.866) and a weak correlation of the instrument with the positive PANSS (r = 0.292) thus characterizing adequate convergent and discriminant validities, respectively. The exploratory factor analysis identified two distinct factors, namely, motivation/pleasure and emotional expressivity, accounting for 68.63% of the total variance.

**Discussion:**

The study shows that the Brazilian version of the BNSS has adequate psychometric properties and it is a reliable instrument for the assessment of negative symptoms in schizophrenia, either for clinical practice or research.

